# TREK channels in Mechanotransduction: a Focus on the Cardiovascular System

**DOI:** 10.3389/fcvm.2023.1180242

**Published:** 2023-05-23

**Authors:** Salvador Herrera-Pérez, José Antonio Lamas

**Affiliations:** ^1^ Laboratory of Neuroscience, CINBIO, University of Vigo, Vigo, Spain; ^2^ Laboratory of Neuroscience, Galicia Sur Health Research Institute (IISGS), Vigo, Spain

**Keywords:** TREK, mechanobiology, cardiovascular system, heart, mechano-feedback

## Abstract

Mechano-electric feedback is one of the most important subsystems operating in the cardiovascular system, but the underlying molecular mechanism remains rather unknown. Several proteins have been proposed to explain the molecular mechanism of mechano-transduction. Transient receptor potential (TRP) and Piezo channels appear to be the most important candidates to constitute the molecular mechanism behind of the inward current in response to a mechanical stimulus. However, the inhibitory/regulatory processes involving potassium channels that operate on the cardiac system are less well known. TWIK-Related potassium (TREK) channels have emerged as strong candidates due to their capacity for the regulation of the flow of potassium in response to mechanical stimuli. Current data strongly suggest that TREK channels play a role as mechano-transducers in different components of the cardiovascular system, not only at central (heart) but also at peripheral (vascular) level. In this context, this review summarizes and highlights the main existing evidence connecting this important subfamily of potassium channels with the cardiac mechano-transduction process, discussing molecular and biophysical aspects of such a connection.

## Introduction

While great strides have been made in understanding the molecular mechanisms of touch (1–3), cardiovascular mechanotransduction remains a complex and enigmatic process that is not yet fully understood. This review highlights the critical importance of mechano-sensitivity in maintaining proper cardiovascular function, and the challenges associated with elucidating its underlying mechanisms.

Multiple elements can sense the different mechanical forces affecting the cellular body, for example, elements of the extracellular matrix such as integrins, elements of the cytoskeleton, G-protein-coupled receptors or different ion channels (4). This review is focused on TREK channels, a subfamily of the two-pore-domain potassium (K2P) channels encoded by genes named KCNK, which are capable of detecting mechanical stimuli altering their opening and closing kinetics. These mechano-sensitive ion channels are membrane proteins that allow cells to respond and adapt to physical forces (5), playing a crucial role in mechano-transduction processes (6, 7). Mechanical forces are fundamental in cardiovascular biology, however, the mechanisms that support this physiological process have yet to be elucidated. In this sense, the link between electrical stimulation and mechanical contractions is widely established, and the mechanism by which an electrical stimulus produces muscle contraction is widely accepted (8). On the contrary, the process by which mechanical forces can influence the electrical properties (mechano-electric feedback) of the cardiovascular cells is still poorly understood (9, 10). Mechano-electric feedback is one of the most important subsystems that operate within the cardiovascular system (11), it can be defined as the process by which mechanical stimuli are converted into electrical signals and plays a key role in the functioning of cardiovascular homeostasis (2, 12, 13). In the heart, different mechano-sensitive structures have been identified, with myocytes being the most relevant (14), while at peripheral level, smooth muscle fibers (present in veins and arteries) are the main elements.

Roughly speaking (without considering chloride channels) ion channels can be separated into two categories. When activated, certain channels, regardless of their selectivity, can either depolarize or hyperpolarize the cell membrane. Applying this idea to the mechanobiology context, these families are known as depolarizing non-selective cationic channels and hyperpolarizing potassium selective channels. In this context, TRP and Piezo channels are part of the first category. They are a nonselective Na^+^, Ca^2+^ (among others) conductors. TRP channels are usually considered as dominant elements in mechano-sensitivity (15) and they are part of the mechanosensitive non-selective cardiac current family (16–19). However, they have been shown to be insensitive to membrane stretch (20) and are not considered primary mechanotransducers (21). Piezo channels are also considered to be transducers of mechanical stimuli and are widely expressed in the cardiovascular system (22). and they could work like baroreceptors (23) even during cardiac development (24). The second category is made up of TREK channels (TREK-1, TREK-2 and TRAAK) and they are probably the only mechanically-gated potassium channels playing an important role in the process of mechanical transduction (25). Given their widespread expression throughout the cardiovascular system (26), these channels are emerging as potential contributors to cardiac mechano-electrical feedback and mechano-associated pathologies. Thus, we reviewed the evidence supporting this possibility.

## Mechano-regulation of TREK channels

MS ion channels can be activated by two different mechanisms. The mechanism called tethering needs several cytoskeletal proteins as scaffold proteins to activate the mechano-sensor, this is the case of TRP channels (27). The other mechanism implies the activation of the channels by the tension in the bilayer itself, without the need for other cellular structures, in this group are TREK channels, see (28) for controversial.

**Figure 1:**
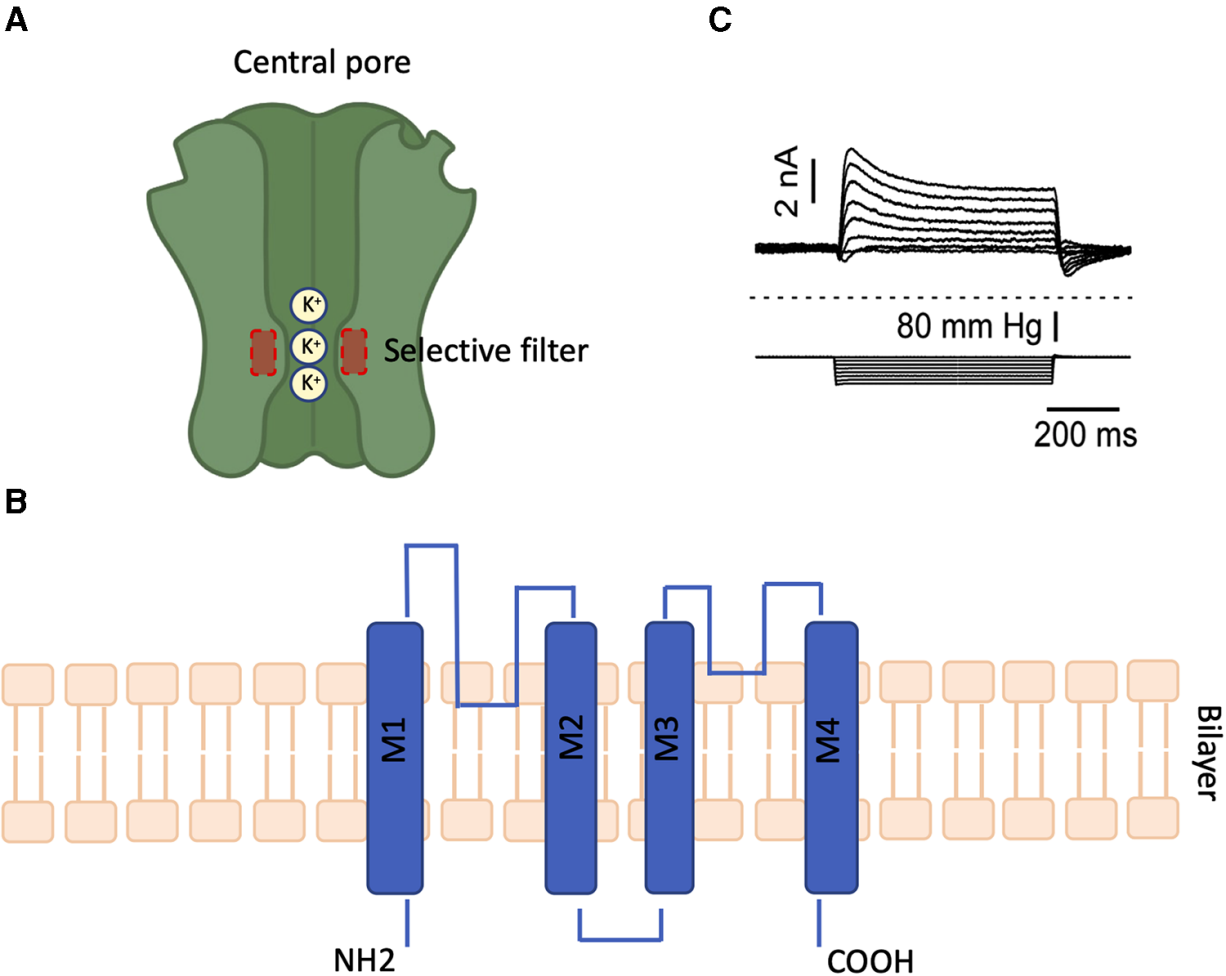
(**A**) view of the TREK structure in conventional configuration. Red dotted lines indicate the selective filter. (**B**) Topological model proposed for K2P channels, each subunit has two pore forming domain (P loops) and four transmembrane domains (denoted M1-M4). (**C**) Representative response to a mechanical stimuli of TREK showing that it has minimal desensitization in the inside-out configuration. Adapted from (32, 33).

The molecular mechanism underlying the sensitivity of TREKs to membrane deformation induced by mechanical forces has been extensively investigated, stating that cellular integrity is not essential for mechanical channel activation (7, 29), indeed TREK channels are regulated by a mechanism called “selective filter” (30, 31) (see Figure 1). This consists in a change of conformation in the narrow zone of the pore that regulates the flow of ions, similar to the C-type blockade studied in voltage-dependent potassium channels such as inward rectifier potassium (Kir) channels (34, 35). It has been shown that with the pore closed, the helical protein structures would not interfere with the passage of ions (36), contrary to what occurs in most potassium channels (37–39). Although this selective filter mechanism is widely accepted (36, 40, 41), there are still many open questions (42) and other gating mechanisms could be present and activated depending on the stimulus (43, 44).

Two states, called “Up” and “Down”, have been described for TREK channels and although in both states the pore is open, it has been suggested that only the Up state can be considered conductive and that in the Down one the conductivity is residual (36, 45). It has been shown that TREK channels can sense mechanical forces directly through the bilayer and it has been demonstrated that TREK channels have located the mechano-gate in the selectivity filter (46, 47). Thus when the membrane is stretched there is a conformational change in the channel's selective filter that favors the entry into the Up state, more conductive when compared with the Down state, notwithstanding this theory has generated some controversy (48). As mentioned above, two mechanisms enable channels to perceive mechanical forces: direct (Piezo and TREK channels) and indirect (TRP channels). In addition to experimental conditions, while the mechanism underlying the mechanosensitivity of TRP channels is well-established (17, 21, 27), it is apparent that membrane deformation can also bring about mechanical changes in different cytoskeleton proteins, which can contribute to the feedback of tension in the bilayer. Therefore, these mechanisms may not be entirely separate and could potentially complement each other under physiological conditions. For instance, some studies have demonstrated that Piezos are solely responsive to shear stress (frictional force) (49, 50), but not to stretch. Furthermore, Piezos can interact with other MS ion channels like TRP channels (51, 52) which may conceal their behaviour under certain experimental conditions, leading to further variability.

## Role of TREK channels in the cardiovascular system

As we have recently reviewed, TREK-1 is the most expressed TREK channel in heart, both in neuronal and non-neuronal tissue, including the sinoatrial node, cardiomyocytes and purkinje fibers (26). Several studies have shown how TREK-1 is extensively expressed in heart using molecular techniques, including qRT-PCR and WB (53, 54). Confocal imaging also showed TREK-1 arranged in longitudinal stripes at the surface of the cardiomyocytes in rats (55). Consistently, whole-cell patch-clamp electrophysiological recordings have shown clearly the presence of a potassium current conducted by TREK channels in cardiac cells in both murine and human (56–58). In summary, the presence of TREK-1 in the heart tissue of various mammals including rodents and humans has been widely demonstrated (Table 1). However, the other two members of the TREK subfamily (TREK-2 and TRAAK) have been poorly localized (32, 64–68).

**Table 1:** Non-systematic but representative summary of the presence of TREK channels in the cardiovascular system.

Protein	Tissue	Method	Reference
TREK-1	h/r/mMyocites	PCR; RT-PCR; Wb; IHC	(46, 59, 60)
TREK-1	h/r/mSA Node	Wb; PCR	(57, 59)
TREK-1	rVentricle	PCR; Wb	(46, 61)
TREK-1	rEndothelial cells	RT-PCR	(62)
TREK-2	rHeart; rVentricular	RT-PCR	(53)
TREK-2	myocytes	RT-PCR	(63)
TRAAK	rEndothelial cells	RT-PCR	(62)

*r:* Rat; *m*: Mouse; *h*: Human; IHC: Immunohistochemistry; Wb: Western Blot; PCR: Polymerase Chain Reaction: RT-PCR: Reverse Transcriptase–Polymerase Chain Reaction.

**Figure 2:**
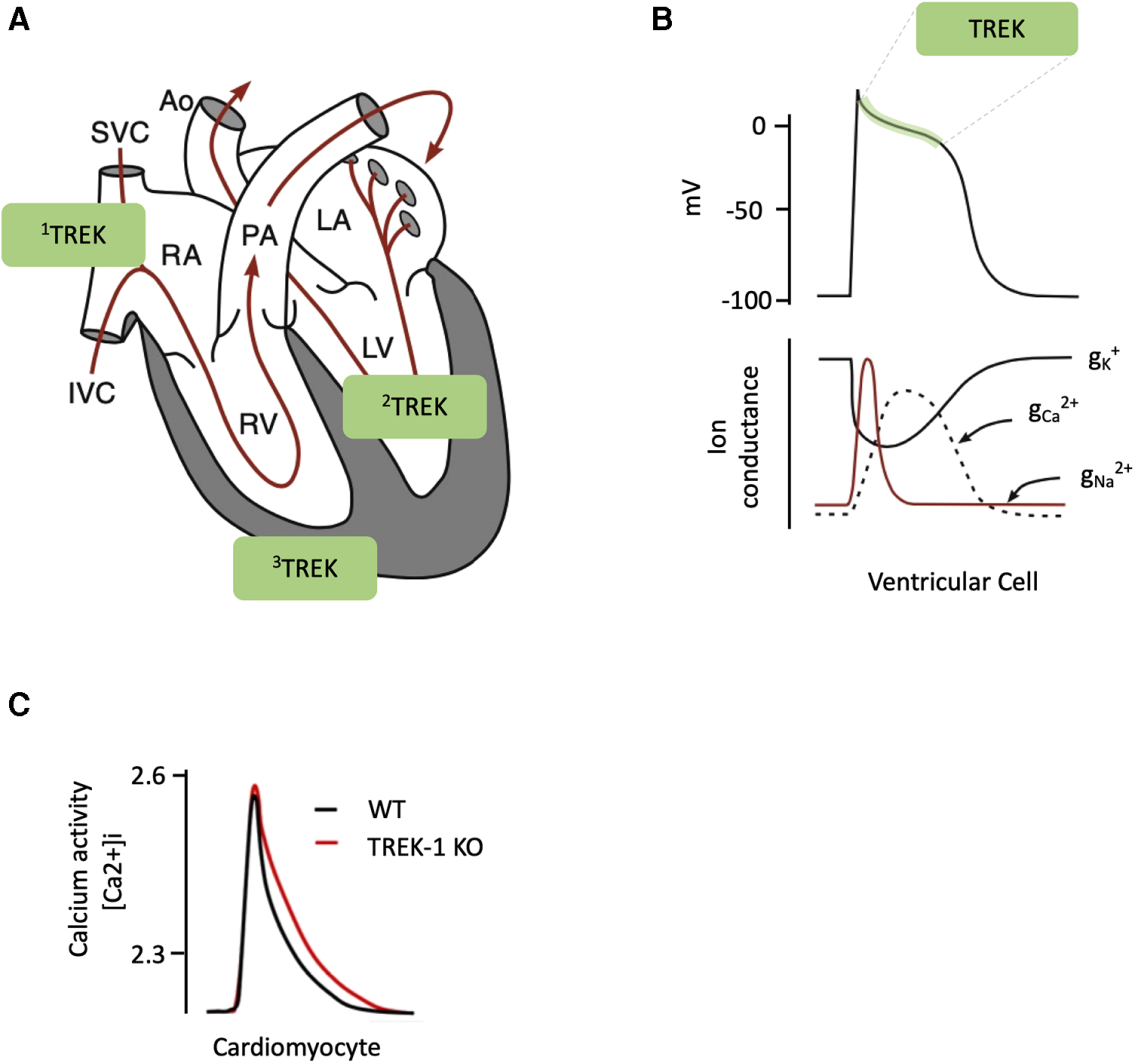
(**A**) representative diagram of a heart showing blood flow (red line) and the different regions of interest: right atrium (RA); superior vena cava (SVC) and inferior vena cava (IVC); right ventricle (RV); pulmonary artery (PA); left atrium (LA); left ventricle (LV) and aorta (Ao). The TREK channels have been schematically represented as 1: sinoatrial node, 2: conduction system (Purkije fibres) and 3: muscle cells. (**B**) Shape of a typical action potential (top) and the conductances that generate it (bottom). Indicating the area where TREK channels are most likely to be involved (green shaded area). (**C**) Effect of TREK channel removal on intrathecal calcium [Ca^2+^]i activity in mouse cardiomyocytes. Adapted from (56, 73).

From a functional point of view, TREK-1 plays a critical role in countering the depolarizing effect of mechano-activated cationic currents, contributing to stimulation-activated central (heart) feedback mechanics in the cardiovascular system (69). TREK-1 channels also have a potential role in regulating the normal activity of sinoatrial node-hosted pacemakers by preventing the occurrence of ventricular extrasystoles (55, 70). Inhibition of TREK-1 channels *via* PKA during sympathetic stimulation may decrease transmural dispersion of repolarization and prevent the occurrence of arrhythmias (58), indicating that TREK-1 may have an essential function in the cardiac conduction system (71). In cardiomyocytes, the refractory period is critical in preventing premature excitation and arrhythmias. The duration or amplitude of the action potential depends on a delicate balance between inward-potassium and outward currents during the action potential plateau. TREK-1, as well as BKCa (large conductance K^+^ channel, both voltage and calcium-gated) or KATP (ATP-sensitive potassium) channels, are the main candidates encoding the cardiac stretch-activated potassium current (72). However, in contrast to TREK-1, in the human heart, BKCa and KATP channels are poorly expressed, making TREK-1 the primary candidate for encoding cardiac stretch-activated potassium currents in different species, with a single channel conductance of approximately 100 pS (9, 32, 58). These results suggest a clear role for TREK-1 in the repolarization phase of the cardiac action potential (Figure 2).

Finally, the variable distribution of TREK-1 in both endothelial and smooth muscle cells across different regions of the heart could facilitate precise regulation of the depolarization wave that initiates cardiac contraction (74). For example, TREK-1 is less present in myocytes of the epicardium of adult rats than in endocardial cells (12).

At the same time, TREK channels play an important role in cardiovascular diseases (47), so that its experimental withdrawal is expected to be pro-arrhythmic (75). TREK-1 has been associated with reduced right atrial channel expression in Atrial fibrillation (AF) models (46). AF is the most common cardiac arrhythmia and results from shortening of atrial effective refractory periods and from a localized deceleration of intra-atrial conduction (76–78). In this context, we recently have shown that verapamil (a class IV antiarrhythmic drug used in pathological conditions such as chronic angina pectoris, cardiac arrhythmias or hypertension) reduces the TREK-1 activity (79). TREK-1 channels may have a role in other pathophysiological situations, during ischemia, when purinergic agonists such as ATP cause the release of arachidonic acid (AA) (80), which lowers intracellular pH, the change of pH/AA can be detected by TREK-1 (63) and could contribute to electrophysiological disturbances in the cardiac mechano-electric feedback (81). TREK-1, plays a protective role against ischaemia-induced neuronal damage and has been shown to play a critical role in cardiac injury and during remodelling after myocardial infarction. Moreover, in TREK-1 KO animals, TREK-1 increases infarct size induced in experimental models, leading to greater systolic dysfunction than its wild-type counterpart, so that activation of TREK-1 may be an effective strategy to provide cardioprotection against ischaemia-induced damage. In addition, a study on the role of TREK-1 in the control of cardiac excitability found that TREK-1 is essential for normal sinoatrial node cell excitability and serves as a potential target for selectively regulating sinoatrial node cell function (56, 57).

Also at peripheral level, the expression pattern of TREK channels in the vascular system has been widely demonstrated. For example, TREK-1, TREK-2 and TRAAK have been detected in various vascular structures such as the pulmonary and femoral artery and the cerebral arteries in both murids and humans, suggesting a putative role for these channels in the vascular system, particularly for TREK-1 (81). Through WB and RT-PCR, TREK mRNA was detected in rat mesenteric and pulmonary arteries (62) and TREK-1 has been suggested to influence mechanically induced endothelial signalling by modulating nitric oxide production (69). In heterologous systems it has been shown that the presence of treprostinil (a tricyclic benzidine analogue of PGI_2_ used for treatment of pulmonary arterial hypertension) was able to inhibit TREK-1 and TREK-2, supporting the idea that TREK-1 could contribute to the cardiac mechano-electric feedback with a hyperpolarizing current in response to mechanical forces in the vascular system.

## Concluding remarks and perspectives

Two types of currents activated by mechanical stimulation operate in the heart, on the one hand a depolarizing non-selective cationic current and on the other hand a hyperpolarizing outward current mainly transported by potassium. Despite originating some controversy (20), the family of depolarizing non-selective cationic current is mainly composed of TRP and Piezo channels (19, 20), and responds with a wide depolarizing current that occurs mainly in the sarcolemma. Stretch-activated potassium currents are primarily driven by TREK channels, which play an important role in cardiac mechano-electrical feedback, both at the cellular level (e.g., presence in principal cells such as pacemakers and cardiomyocytes) and at the system level through their involvement in the regulation of heartbeat force and rate (6, 19, 82). Overall, there is now some evidence for the ability of TREK channels to control the electrical activity of the heart through central mechano-electrical feedback (19).. It has been proposed that the main function of TREK-1 is to counteract the depolarising effect induced by currents activated by mechanical stimuli, thus contributing to central mechano-electric feedback in the cardiovascular system (55, 69) and controlling, at least in part, the early repolarisation phase and action potential transfer through the ventricular conduction system. Finally, as mentioned above, it appears that TREK channels, especially TREK-1, may play a role in nodal pacemaker activity.

The strong presence of TREK-1 could also indicate a possible role in the mechanical control of the electrical activity of the vascular periphery. However, other players must be considered in the mechano-electric feedback process. Recent work has investigated the role of Piezo 1 channels in the development of cardiac hypertrophy, showing how activation of calcium/calpain signalling through the Piezo 1 pathway contributes to the development of cardiac hypertrophy in murine models. Furthermore, Piezo 1 is a cardiac mechano-sensor that is activated in response to cardiac overload in adult animals, which in turn initiates the myocardial hypertrophic response. On the other hand, it has also been shown that Piezo 1 activation in response to mechanical stimuli triggers chemical signals that contribute to the physiological response of the heart to mechanical stress (83–85). These findings undoubtedly support the relevant role that Piezo channels may play in both mechano-electric feedback and cardiac pathophysiology.

In summary, TREK channels are involved in the regulation of mechanical forces both centrally and peripherally in the cardiovascular system. It should be noted that there may be other currents at play that could contribute to or even counteract TREK activity. More important, recent data have shown that antiarrhythmic drugs can interact with mechanically-gated TREK channels. There is enough evidence supporting the hypothesis that potassium outward currents driven by TREK channels play an important role not only in the normal functioning of the cardiovascular system, where its mechanical sensitivity plays a central aspect, but also in some relevant pathologies such as AF and other cardiac conditions. The expression of TREK-1 channels in the ventricle exhibits regional heterogeneity, similar to that observed in mechano-electrical feedback under physiological conditions. Consequently, this regional variability in TREK-1 channel expression has the potential to modulate mechano-electrical feedback, resulting in altered repolarization of the action potential and consequent arrhythmogenic effects (86). Although in this review we have focused on the possible role of TREK channels in cardiac mechano-electric feedback as well as their possible role in the phytopathology of the heart, there is no doubt that other MS ion channels such as Piezo channels must be taken into account in the explanation of the molecular mechanism underlying cardiac mechano-electric feedback.

Presently, a significant limitation exists in the investigation of the possible role of TREK channels in the mechano-electric feedback process due to the lack of identified specific TREK channel blockers. Nonetheless, a considerable body of evidence supports the proposition that these channels are unequivocally responsible for potassium current in response to mechanical stimuli, and given their abundant expression in the cardiovascular system, it is highly probable that they are fundamental in the feedback process. Furthermore, as previously remarked, there is clear evidence indicating that TREK channels have a relevant role in cardiac pathophysiology. However, despite the extensive evidence of TREK channel presence in various regions of the cardiovascular system, including sympathetic innervation, it is presently unknown if these channels are also present in the intracardiac ganglion, which is responsible for parasympathetic control of cardiac activity. Moreover, as the pharmacology of TREK channel usage progresses, it is conceivable that more appropriate experimental designs can be employed to elucidate the relationship between TREK channels and mechano-electric feedback more clearly.
